# Design and implementation of informatization for unified management of stroke rehabilitation in urban multi-level hospitals

**DOI:** 10.3389/fnins.2023.1100681

**Published:** 2023-02-15

**Authors:** Lihua Huang, Lan Jiang, Yiming Xu, Yanhong Ma

**Affiliations:** Shanghai Sixth People’s Hospital, Shanghai Jiao Tong University School of Medicine, Shanghai, China

**Keywords:** stroke rehabilitation, three-level rehabilitation, unified management, informatization, hierarchical diagnosis and treatment

## Abstract

**Background:**

With the aging of the population, the prevalence and incidence of stroke in China are increasing every year. China advocates the establishment of a three-level medical service system for stroke rehabilitation, but it lacks uniform information management among all levels of medical institutions.

**Objective:**

To achieve unified management of stroke patient rehabilitation in multilevel hospitals in the region through informatization construction.

**Methods:**

The need for informatization of three-level stroke rehabilitation management was analyzed. Then, network connections were established, and a common rehabilitation information management system (RIMS) was developed for all levels of hospitals to enable daily stroke rehabilitation management, inter-hospitals referral, and remote video consultation. Finally, the impact on the efficiency of daily rehabilitation work, the functioning and satisfaction of stroke patients were investigated after implementing the three-level rehabilitation network.

**Results:**

One year after implementation, 338 two-way referrals and 56 remote consultations were completed using RIMS. The stroke RIMS improved the efficiency of doctors’ orders, reduced therapists’ time to write medical documents, simplified statistical analysis of data and made referrals and remote consultations more convenient compared to the traditional model. The curative effect of stroke patients managed by RIMS is better than that of traditional management. Patient satisfaction with rehabilitation services in the region has increased.

**Conclusion:**

The three-level stroke rehabilitation informatization has enabled the unified management of stroke rehabilitation in multilevel hospitals in the region. The developed RIMS improved the efficiency of daily work, improved the clinical outcomes of stroke patients, and increased patient satisfaction.

## 1. Introduction

Stroke is the second leading cause of death worldwide, accounting for 1 in 10 deaths (Kim and Johnston, 2013), and the prevalence, incidence, and mortality rates are higher in developing countries than in developed countries (Mehndiratta et al., 2014). According to the Report on National Health Commission (2020), stroke remains the number one cause of disability and mortality in China (Chao et al., 2021a). The National Epidemiological Stroke Survey of China (NESS-China) estimated 11 million cases of stroke, 2.4 million new strokes, and 1.1 million stroke-related deaths in China each year (Wang et al., 2017), causing a huge economic burden on families and society (Zhao et al., 2022).

Stroke rehabilitation is a critical measure to reduce disability and improve the quality of life, which is an integral part of the treatment system for cerebrovascular disease (Langhorne et al., 2011). In China, the Ministry of Health China Stroke Prevention Project Committee (CSPPC) was established in 2011 (Chao et al., 2021a). The officials advocate the establishment of a three-level stroke rehabilitation service system (Chen et al., 2010), with primary rehabilitation for acute and sub-acute stroke rehabilitation in tertiary hospitals or tertiary rehabilitation hospitals, secondary rehabilitation for convalescent phase of stroke in secondary hospitals or secondary rehabilitation hospitals, and tertiary rehabilitation for chronic phase of stroke in community health service centers or township health centers or families. Although the three-level stroke rehabilitation system has established recommended referral criteria and treatment protocols (Xu et al., 2021), the lack of uniform management practices across all levels of care may delay the treatment due to poor referral, indicating that coordinating the treatment of stroke patients between the acute, convalescent, and chronic phases is essential (Kinoshita et al., 2021).

The development of information technology facilitates uniform stroke rehabilitation management in hospitals at all levels in the region. Xuhui District in Shanghai has seized the opportunity of smart city construction to connect all levels of hospitals in the region through network- and design-dedicated rehabilitation management software. Also, tertiary rehabilitation referral criteria and recommended rehabilitation treatment protocols applicable to the region were developed. This study aimed to analyze the need for the informatization of a three-level rehabilitation network for stroke and present the hardware and software’s design, implementation, and efficiency.

## 2. Materials and methods

### 2.1. Analysis of the informatization needs of three-level stroke rehabilitation management

First, an information management system for stroke rehabilitation that can be used in primary, secondary, and tertiary hospitals needs to be developed. A total of 16 hospitals were involved in this project in Xuhui District, Shanghai. Among these, 13 were primary hospitals (i.e., Community Health Service Centers), including Xujiahui Street, Hunan Street, Tianping Street, Xietu Street, Longhua Street, Fenglin Street, Tianlin Street, Caohejing Street, Kangjian Street, and Hongmei Street, two were secondary hospitals, including Xuhui Central Hospital, and Xuhui Dahua Hospital, and one was a tertiary hospital, Shanghai Sixth People’s Hospital. In the three-level rehabilitation management of stroke, hospitals at different levels treating stroke in different phases lead to variations in the types and difficulties with respect to rehabilitation equipment, rehabilitation technicians, and rehabilitation treatment technologies. Therefore, during the software design, the rehabilitation evaluation and treatment technology in the system should be customized according to the actual situation.

Second, the diagnosis and treatment information of stroke patients need to be shared among different hospitals, such that when stroke patients are referred between hospitals at different levels in the region, historical information on rehabilitation can be shared through downloads. This avoids duplication of tests, examinations, and assessments and provides a basis for the next stage of treatment.

Third, remote video consultation is required. It is common for primary hospitals to request consultations from secondary and tertiary hospitals. Teleconsultations through web-based video conferencing can improve the efficiency of consultations in a time-effective manner.

### 2.2. Design and implementation of a three-level stroke rehabilitation network in the region

#### 2.2.1. Design thinking

Based on the above requirement analysis, in terms of hardware, a data center and multiple subcenters are established using a distributed architecture. All data centers are connected. Each data center does not rely on the general data center to operate independently. In the case of a data request (such as referral and consultation), data exchange can be generated. If one of the network members (Unit A) needs to refer a patient to another network member (Unit B), the patient’s data are uploaded from Unit A to the data center, and Unit B downloads the patient’s data, thus avoiding network congestion caused by frequent synchronization. In terms of software, a common stroke rehabilitation management system for primary, secondary, and tertiary hospitals will be developed, with integrated data synchronization and remote video consultation modules.

#### 2.2.2. Hardware and network connectivity

**FIGURE 1:**
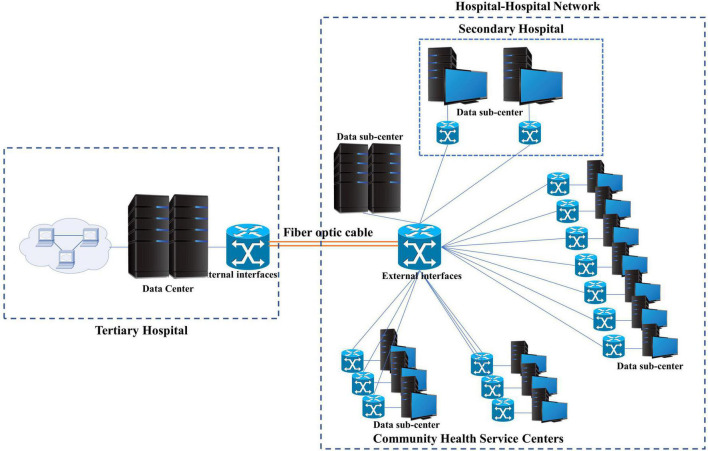
Topology of the three-level stroke rehabilitation network.

As shown in Figure 1, the data center is located in the tertiary hospital, which is connected to the client of the rehabilitation medicine department through the hospital’s internal network and the Hospital-Hospital Network of Xuhui District through an optical fiber. The Hospital-Hospital Network connects 13 primary hospitals (Community Health Service Centers) and two secondary hospitals in Xuhui District.

#### 2.2.3. Software main modules and functions

The same rehabilitation information management system (RIMS) is used in all levels of hospitals. The software is a B/S (browser/server) architecture, wherein the client interacts with the server *via* a web page for data interaction. C# and Net are used for the data center, application service, and client. The server operating system is Windows Server 2008 R2 Enterprise, and the database is Windows SQL Server 2008 + MySQL. The functional modules of the software are as follows.

##### 2.2.3.1. User management

The system divides users into five roles: Doctor, nurse, therapist, administrator, and super-administrator, with each role assigned after appropriate permissions. The doctor’s role is to issue and amend medical orders; the nurse views and bills the orders; the therapist views the medical orders and submits the treatment records; the head of the department and the leader of health administration review the operational data as administrators; the super administration is effectuated by the software designer to make changes or delete the software function modules.

##### 2.2.3.2. Medical records management

The system manages the medical history of all stroke patients in both outpatient and inpatient settings. When a patient needs rehabilitation treatment, medical record information is entered as required, including basic patient and medical history information. The former includes name, gender, age, hospitalization number, and ward and bed number, while the latter includes the patient’s chief complaint, medical history, physical examination, auxiliary tests, and diagnosis.

##### 2.2.3.3. Rehabilitation medical order management

The system categorizes all the rehabilitation technology items carried out in the treatment room. Each rehabilitation technology item contains five sub-items: Technology name, dose, time, site, and frequency, and the items have been set to default values so that doctors can easily issue medical orders. After the doctor submits the orders, the therapist can view the detailed list of orders in the rehabilitation treatment interface and initiate the treatment according to the orders. The system can automatically record the relevant parameters of the rehabilitation treatment, such as the operator, operation time, and actual dose. The previous medical orders could be copied automatically when the patient re-visited or modified and submitted according to the situation, which greatly improves the efficiency of the re-visit.

##### 2.2.3.4. Rehabilitation assessment management

The system provides mandatory and optional assessments for stroke, which can be completed by both doctors and therapists. The assessment results can be viewed in the same interface, such that the patient’s condition can be understood and follow-up treatment can be planned.

##### 2.2.3.5. Staging referrals

Referrals are made between hospitals in accordance with the criteria listed in Table 1. When a referral is made, the system uploads the treatment information of the patient to the data center, and the receiving hospital downloads the patient information. The downloaded treatment information is presented as a report.

**TABLE 1:** Staged stroke referral criteria.

Stages of stroke	Acute and sub-acute phases	Convalescent phases	Chronic phases
Referral Hospitals	Department of Rehabilitation Medicine in a tertiary hospital or a tertiary rehabilitation hospital	Department of Rehabilitation Medicine in a secondary hospital or a secondary rehabilitation hospital	Community Health Center or Home
Referral criteria	Either of the following appears: • Stroke recurrence. • The presence of progressive cerebral edema, severe lung infection, urinary tract infection, sepsis or severe pressure sores. • Impaired consciousness or increased functional impairment. • Multiple organ failure occurs.	The following conditions are all met: • Vital signs are stable. • Neurospecialist treatment finished. • Stroke-related clinical laboratory tests are generally normal or stable. •After receiving early rehabilitation diagnosis and treatment, there are still serious dysfunctions or complications, such as consciousness or cognitive impairment, tracheotomy, acute myocardial infarction, dysphagia, etc.	The following conditions are all met: • Vital signs are stable. • Stroke-related clinical laboratory tests are generally normal. • No serious complications or comorbidities. • Mild functional impairment is present.

##### 2.2.3.6. Teleconsultation

**FIGURE 2:**
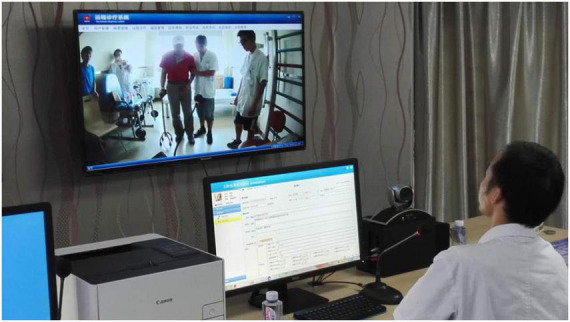
The expert was using the system for remote consultation.

This module initiates a remote video conference when activated. In addition to video and voice communication, a separate window displays the patient’s medical history, history of examination, and treatment for the remote specialist’s reference in the video conference, which helps the specialist to make an accurate diagnosis and give treatment advice (Figure 2).

##### 2.2.3.7. Customization of rehabilitation service items of hospitals at all levels

**FIGURE 3:**
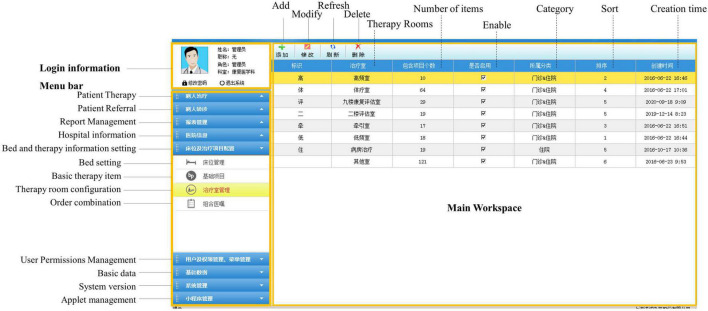
Customized therapy items in rooms.

For the system to be used in the rehabilitation medicine departments of all levels of hospitals, the rehabilitation items need to be personalized. Under super administrator privileges, hospitals at all levels can configure the number of treatment rooms, and the rehabilitation technology programs were included according to their actual needs (Figure 3) or set default values for the dose, time, and site of each rehabilitation technology item.

##### 2.2.3.8. Research support

The unified patient information data facilitates the collection of data. The researchers can use the system for retrospective analysis and also for the evaluation of the efficacy of new technologies.

#### 2.2.4. Network information security

Each hospital has a Department of Information Management responsible for network information security. Clients in the Department of Rehabilitation Medicine are prohibited from connecting to external storage devices for possible information leakage, and communication between sub-centers in each hospital is protected by hardware firewalls.

### 2.3. Evaluation of the effectiveness of a three-level stroke rehabilitation network in the region

After 1 year, we evaluated the effectiveness of the three-level stroke RIMS in terms of daily work efficiency, the function of stroke patients and patient satisfaction. The impact of RIMS on daily work efficiency was studied through face-to-face interviews. The face-to-face interviews were conducted to collect information on the experience of using RIMS, including the time taken to give medical orders at the initial consultation, the time taken to give medical orders at follow-up consultations, the time taken by the therapist to write the medical document, the convenience of statistical analysis of treatment data, the convenience of referral, the convenience of teleconsultation (7-point Likert scales were used for convenience, with 7-points representing very good convenience) and the problems that needed improvement. The impact of RIMS on the function of stroke patients and patients’ satisfaction has been published in Chinese journals (Yuan et al., 2016; Li et al., 2019) and the findings are cited and presented below.

### 2.4. Statistical analysis

All data were analyzed using SPSS 22(SPSS, IBM Corp., USA)The data of time and grade obtained from face-to-face interviews were reported as the mean and standard deviation, and analyzed by paired *t*-test. The satisfaction of patients before and after the project was compared using Ridit analysis. *P*-value < 0.05 and 95% confidence interval were used to indicate statistical significance.

## 3. Results

**FIGURE 4:**
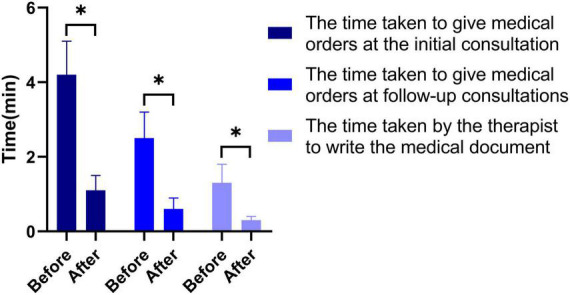
Comparison of the time spent by doctors and therapists on daily medical records before and after project implementation. *P < 0.05.

**FIGURE 5:**
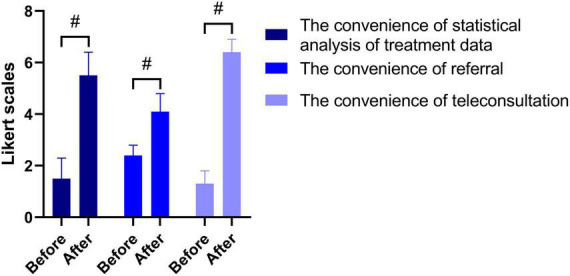
Comparison of the convenience of daily work before and after project implementation. ^#^*P* < 0.05.

A total of 46 clients in one tertiary hospital, two secondary hospitals, and 13 primary hospitals have installed RIMS and accessed the network, and the rehabilitation treatment of both outpatients and inpatients with stroke was managed using RIMS. One year after the implementation, 338 two-way referrals and 56 remote consultations were completed with the software. A total of 32 people were interviewed. The results show that after the use of stroke RIMS, the efficiency of doctors’ orders is improved (*P* < 0.05), the time for therapists to write medical documents is reduced (*P* < 0.05) (Figure 4), statistical analysis of data is simplified (*P* < 0.05), and the referral and remote consultation are convenient compared to the traditional work mode (*P* < 0.05) (Figure 5).

To understand the impact of a regional three-level rehabilitation network on the self-care ability and quality of survival of stroke patients, Yuan et al. (2016) from one of the network member units conducted a retrospective clinical study. The study divided 90 stroke patients into the network and conventional groups, respectively. A total of 60 patients in the network group were treated according to the three-level stroke rehabilitation program, while 30 patients in the conventional group were rehabilitated according to the conventional methods. The results showed that after 6 weeks of intervention, the scores of Modified Barthel Index and Stroke-Specific Quality of life in both groups were significantly higher than those before the treatment (*P* < 0.05), and those in the network group were significantly higher than those in the conventional group (*P* < 0.05). This finding showed that the information management of the three-level rehabilitation network for stroke can improve the rehabilitation effect.

**FIGURE 6:**
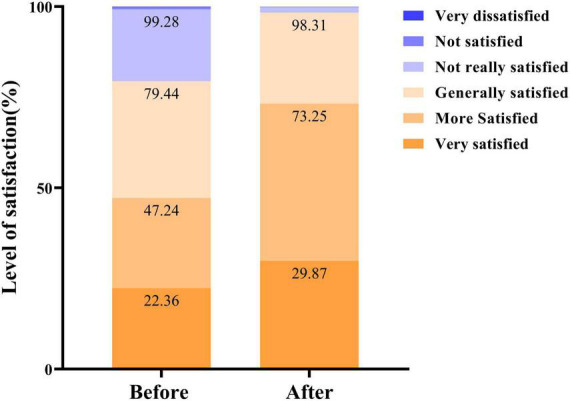
Comparison of resident satisfaction before and after project implementation.

To understand the satisfaction of residents in Xuhui District with three-level stroke rehabilitation, we conducted a questionnaire survey on a sample of six streets in Xuhui District (Li et al., 2019). The results of the research analysis by the independent third-party management consulting organization (ZERO Power Intelligence Group) showed that the overall satisfaction of residents with community rehabilitation services increased from 79.44 to 98.31% (*P* < 0.01) (Figure 6; Li et al., 2019).

## 4. Discussion

The three-level stroke RIMS introduced in this article enabled the unified management of stroke rehabilitation patients in 16 hospitals at different levels in the Xuhui District of Shanghai. Finally, efficient daily work, referral and remote consultation were realized, which improved patients’ satisfaction with rehabilitation services, as well as the clinical outcomes of rehabilitation treatment.

### 4.1. Three-level stroke rehabilitation

With increasing life expectancy, the population ages and living standards are also improved, and the prevalence and incidence of stroke are increasing (Zhao et al., 2022). The development of Stroke Network, Stroke Map, and Stroke Green Channel has reduced stroke mortality (Chao et al., 2021b), but the increase in prevalence has elevated the absolute number of stroke patients and the subsequent cost of stroke care year by year. Health managers have been trying to achieve cost savings *via* clinical pathways without reducing the effectiveness of care.

Previous studies have shown that clinical pathways can significantly reduce the length of hospital stay and the cost of stroke care for patients (Deng et al., 2014; Pilato et al., 2021). The three-level rehabilitation, also known as the regional clinical pathway (Fujino et al., 2014), is a seamless integration of the acute stroke clinical pathway (Stroke Unit), the convalescent clinical pathway, and the chronic clinical pathway (Xu et al., 2021). This facilitates the discharge of stroke patients with significant burdens and allows the tertiary hospitals to accept patients with severe conditions (Van der Cruyssen et al., 2015) and refer discharged patients to the next level of the hospital for appropriate treatment according to their condition, thus saving treatment costs without reducing the treatment effect. Another reason why three-level rehabilitation is possible in China is related to the hierarchy of hospitals. Unlike Western countries, such as the USA and Europe, most cities in China have primary, secondary, and tertiary hospitals corresponding to the acute, convalescent, and chronic phases of stroke. Some Asian countries refer to the second level of three-level rehabilitation as transitional rehabilitation (Leigh et al., 2022), which does not change the fact that they also practice three-level rehabilitation (Kinoshita et al., 2021). In China, many studies have confirmed that the three-level stroke rehabilitation network is beneficial for rehabilitation of stroke patients, and was recommended by the Chinese Stroke Association guidelines for clinical management of cerebrovascular disorders (Zhang et al., 2020).

### 4.2. Informatics-assisted three-level stroke rehabilitation

China’s hospital informatization is the earliest area of medical informatization development and is now widespread in the country. However, one hospital’s informatization is only an information island (Liang et al., 2019), while the regional medical consortium based on the information network is the direction of future development to promote hierarchical diagnosis and treatment. Currently, the construction of regional medical informatization has become the hot spot of medical informatization in China (Zhang et al., 2022).

A key component of regional medical informatics is the collaborative management of common and chronic diseases, such as stroke. However, the current focus of regional medical informatics is on the establishment of pre-hospital stroke networks, which have been established in different countries and regions, such as the Cardiovascular Health and Stroke Strategic Clinical Network (CvHS SCN) in Canada (McIntosh et al., 2019), Emergency Medical Services (EMS) in Korea (Park et al., 2016), the Neurovascular Network of Southwest Bavaria (NEVAS) in Germany (Feil et al., 2021) and the Distributive Stroke Network (DSN) in USA (Holder et al., 2021). In China, although CSPPC has led a Stroke Network that has shown initial success in stroke prevention and emergency care (Chao et al., 2021a), post-stroke three-level rehabilitation informatics is still underdeveloped.

Currently, fewer studies have focused on the role of post-stroke rehabilitation network informatics, and post-stroke rehabilitation informatics has only been mentioned when investigating data such as healthcare costs and length of stay. In the study of the role of regional clinical pathways, Japanese researcher Fujino (Fujino et al., 2014) described the Diagnosis Procedure Combination (DPC) database system they used. The DPC system was a national case-mix patient classification system and consisted of clinical data from 82 university hospitals, which played an important role in the statistical correlation of regional clinical pathways and length of stay. The Korean researcher Leigh (Leigh et al., 2022) referred to the National Health Insurance (NHI) claims database in their review of the Korean stroke transitional and long-term rehabilitation care system. Through this database, researchers were able to investigate information such as inpatient rehabilitation and outpatient rehabilitation patients, and the length of time patients received rehabilitation services, which assisted with statistics such as the cost of rehabilitation. The three-level stroke RIMS developed in this project is not the same as the DPC and NHI. Recording information on the cost of rehabilitation is only one of its functions and the more important function is to record rehabilitation process while providing referral and teleconsultation functions similar to those of the pre-hospital stroke network. This study found that the three-level stroke RIMS not only improved the efficiency of daily stroke management but also improved the satisfaction and clinical outcomes of stroke patients. This observation was consistent with the findings of Xu et al. (2021).

### 4.3. Challenges of informatization

The process of stroke three-level rehabilitation informatization may also encounter some problems. Firstly, the Hospital Information System (HIS) varies across hospitals, and establishing a standard format is difficult. This could be ascribed to the need to manually enter the basic information of stroke patients in the RIMS. Secondly, the network connection may encounter interface restrictions and security restrictions, resulting in the inability to connect to the network. Thus, several meetings were undertaken to communicate and purchase new gateway equipment before it was resolved. Thirdly, the willingness to use the software was low. Some doctors and therapists were accustomed to the original treatment process, thereby using the software less frequently. These users should be informed of “Thorndike’s law,” which is a learning curve for the software, and that although the work efficiency is low in the beginning, it increases significantly when they become proficient.

### 4.4. Deficiencies of the project

Nevertheless, this stroke three-level rehabilitation informatization has some shortcomings. Firstly, there are still flaws in the software design. The software was not designed to gather all the requirements for use at the beginning; for example, some doctors only raised the issue after the project was completed and wanted to add a stroke knowledge database and work reminder functions. Secondly, the RIMS could not be synchronized with the HIS systems of different hospitals. This increased the manual input workload, which seemed to be a problem that could not be solved even at a later stage. Finally, the assessment methods for using RIMS were not established, discouraging the doctors from including all stroke patients.

## 5. Conclusion

The three-level stroke rehabilitation informatization has enabled the unified management of stroke rehabilitation in multilevel hospitals in the region. The developed RIMS can improve the efficiency of daily work and the clinical outcomes of stroke patients, thereby increasing patient satisfaction. This project provides a reference for other regions to establish a unified regional management of chronic diseases.

## Data availability statement

The original contributions presented in this study are included in this article/supplementary material, further inquiries can be directed to the corresponding authors.

## Author contributions

YX: conceptualization, software, and writing – review and editing. YM: methodology and supervision. LH and LJ: investigation. LH and YX: data curation. LJ: resources. LH: writing – original draft preparation. All authors have read and agreed to the published version of the manuscript.
